# Case Report: Deep Brain Stimulation to the Ventral Internal Capsule/Ventral Striatum Induces Repeated Transient Episodes of Voltage-Dependent Tourette-Like Behaviors

**DOI:** 10.3389/fnhum.2020.590379

**Published:** 2021-01-25

**Authors:** Joan A. Camprodon, Tina Chou, Abigail A. Testo, Thilo Deckersbach, Jeremiah M. Scharf, Darin D. Dougherty

**Affiliations:** ^1^Department of Psychiatry, Harvard Medical School, Boston, MA, United States; ^2^Department of Neurology, Massachusetts General Hospital, Harvard Medical School, Boston, MA, United States

**Keywords:** DBS, neuromodulation, MDD, Tourette syndrome, PET, brain stimulation

## Abstract

Deep Brain Stimulation (DBS) is an invasive device-based neuromodulation technique that allows the therapeutic direct stimulation of subcortical and deep cortical structures following the surgical placement of stimulating electrodes. DBS is approved by the U.S. Federal Drug Administration for the treatment of movement disorders and obsessive-compulsive disorder, while new indications, including Major Depressive Disorder (MDD), are in experimental development. We report the case of a patient with MDD who received DBS to the ventral internal capsule and ventral striatum bilaterally and presented with 2 weeks of voltage-dependent Tourette-like symptoms including brief transient episodes of abrupt-onset and progressively louder coprolalia and stuttered speech; tic-like motor behavior in his right arm and leg; rushes of anxiety, angry prosody, angry affect; and moderate amnesia without confusion. We describe the results of the inpatient neuropsychiatric workup leading to the diagnosis of iatrogenic voltage-dependent activation of cortico-subcortical circuits and discuss insights into the pathophysiology of Tourette as well as safety considerations raised by the case.

## Introduction

Deep Brain Stimulation (DBS) is an invasive neuromodulation technique that allows the direct stimulation of subcortical and deep cortical structures following the surgical placement of brain electrodes connected to an implantable battery-powered pulse generator. It aims to act as a neural pacemaker, improving function in diseased neuronal populations, and facilitating therapeutic adaptive changes in brain networks. The exact mechanism of action and optimal therapeutic parameters for each target and pathology are yet to be established, but a significant amount of research in animal models and humans is underway (Herrington et al., 2016).

Following successful engineering and clinical developments in the field of cardiac pacemakers, DBS emerged as an alternative to ablative neurosurgical procedures that provided several advantages: its reversible nature, the capacity to modulate the parameters of stimulation, and its potential for placebo-controlled blinded studies. DBS is approved by the U.S. Federal Drug Administration (FDA) for the treatment of patients with severe and refractory movement disorders (including Parkinson’s disease, Essential Tremor, and Dystonia) or Obsessive-Compulsive Disorder (OCD). Also, several experimental approaches are exploring the use of DBS for other treatment-resistant neuropsychiatric disorders, including Major Depressive Disorder (MDD; Kaur et al., 2013), Gilles de la Tourette Syndrome (GTS; Andrade and Visser-Vandewalle, 2016), and others (Arulpragasam et al., 2013).

Here, we report the case of a patient with MDD implanted with DBS on the ventral internal capsule/ventral striatum (VC/VS) who presented with 2 weeks of Tourette-like symptoms including brief transient episodes of stuttered and progressively louder speech with coprolalia; predominantly right-sided tic-like behaviors; rushes of anxiety, angry prosody, angry affect; and moderate amnesia without confusion. We describe the course of the admission and diagnostic workup, including structural and functional neuroimaging data to support a mechanistic pathophysiological hypothesis and discuss DBS safety considerations raised by the case.

## Methods

### Case Report

**Figure 1:**
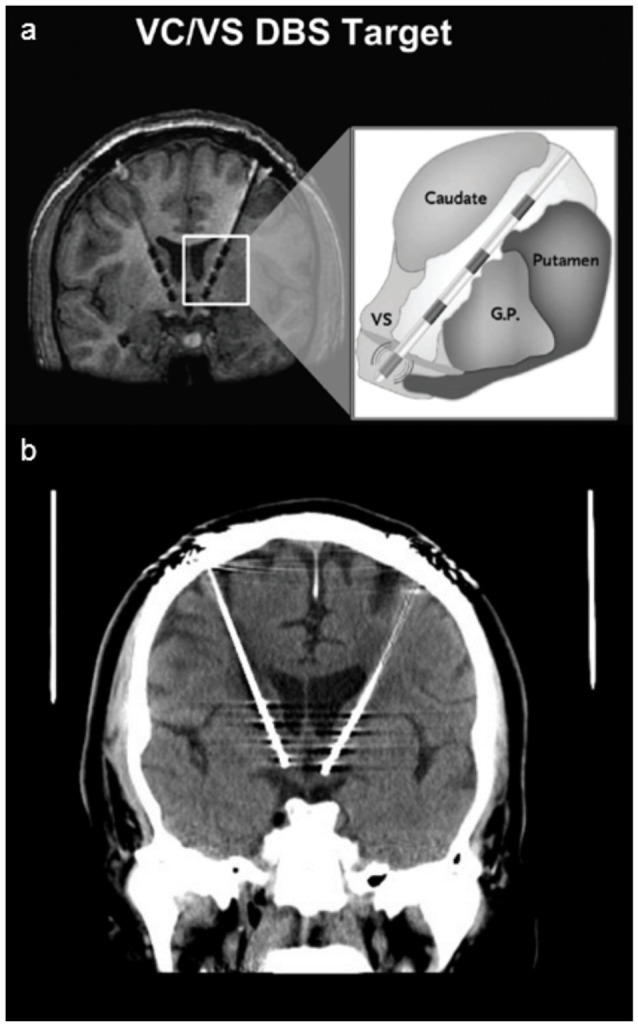
Deep Brain Stimulator. Panel **(A)** shows a schematic of Deep Brain Stimulation (DBS) electrodes placed on the ventral capsule/ventral striatum with four different electrodes placed from ventral (0) to dorsal (3; image used with permission from Medtronics). Panel **(B)** shows the head CT of the patient, revealing the more ventral and anterior position of the left electrode.

The timeline of events can be found in Table 1. A 57-year-old right-handed single Caucasian male with a history of severe, treatment-resistant MDD since the age of 20 and a Deep Brain Stimulator implanted bilaterally in the VC/VS (Figure 1A) 32 months before admission, presented to the Emergency Room of the Massachusetts General Hospital complaining of 2 weeks of brief transient episodes, lasting 20 s to 2 min, of abrupt-onset and progressively louder coprolalia; tic-like motor behavior in his right arm or leg; rushes of anxiety, angry prosody, angry affect; and moderate amnesia without confusion. No obsessions or compulsions were reported. The patient’s history also includes Panic Disorder without Agoraphobia in full sustained remission, Alcohol Dependence, Hypercholesterolemia, Hypertension, Peptic Ulcer Disease, and Lumbar Spondylosis.

**Table 1:** Timeline of events.

Time	Event	Description
35 years before admission	The onset of Major Depressive Disorder (MDD) and panic attacks.	Since the age of 20.
30 years before admission	Panic attacks resolved with medication.
~28 years before admission	Patient report of alcohol abuse and dependence.	Patient attempt to “self-medicate” mood.
7 years before admission	Vagus Nerve Stimulator (VNS) implanted for treatment-resistant MDD.	
3 years before admission	Received Electroconvulsive Therapy (ECT).	Tolerated 2 sessions with mild post-ECT confusion; encephalopathic, confused and disoriented for 5 days after 3rd session. ECT stopped.
2 years before admission	VNS device explanted.	Due to lack of efficacy.
2 years before admission	Deep Brain Stimulator (DBS) implanted for treatment-resistant MDD.	Bilaterally in ventral internal capsule/ventral striatum; positive response for ~2 years.
~1–2 years before admission	Patient scanned with Positron Emission Tomography (PET).	In the context of a neuroimaging research study; inhaled radiolabeled CO2 molecules. Images acquired while stimulating at different DBS electrode positions.
16 weeks before admission	Depressive symptoms worsened; resumed drinking up to four mixed drinks per day.	
11 weeks before admission	Radioablative treatment for lumbar spondylosis.	
2 weeks before admission	The onset of the patient presenting complaints.	Brief episodes (20 s-2 min) of garbled nonsensical speech with paraphrasias, neologisms, agrammatism, coprolalia, rushes of anger and anxiety without panic symptoms, motor automatisms on the right arm and right leg.
1 day before admission	The patient contacted the psychiatric neurotherapeutics team with presenting complaints.	The patient advised to turn the DBS device off and present it to the clinic.
Admission	The patient was seen at the outpatient clinic, referred to the Emergency. Department, admitted to Neurology service.	DBS device turned off, normal neurological exam.
During admission		Negative work up for TIA/stroke with CT of the brain, CTA of brain and neck, TTE, and 24 h Holter.
		Normal limits for lipid panel, hemoglobin A1C, Vitamin B12, Thiamine, TSH, urine drug screen, blood alcohol level.
		Negative work up for seizures with EEG when DBS device was off (and later on).
		Two possible but unclear events with no epileptiform activity during 24 h long-term monitoring EEG.
	DBS device turned on at original settings.	Short episodes of progressively faster and louder stuttering with coprolalia, neologisms, agrammatism, paraphrasias, right-sided tic-like motor automatisms, ego-dystonic “rush” of physical and psychological activation; the patient had minimal memory of these events after they occurred.
	The psychiatric neurotherapeutics team consulted.	The voltage of the DBS device increased from 7 to 8 V with no changes, then increased to 8.5 V. The event started immediately and ceased when the voltage was reduced to 7 V.
Discharge	Patient discharged and scheduled to follow up with neurotherapeutics team.	
1 day after discharge	The patient presented back at the outpatient clinic.	DBS device turned on. Decreased pulse width to 90 μs of the left electrode, voltage left at 9V. The Patient reported no side effects and left with a safety plan.
3 weeks after discharge	The patient called the neurotherapeutics team.	Reported having a few brief similar episodes; advised to turn the stimulator off.
	The patient returned to the outpatient clinic.	Outpatient EEG with DBS device on at a higher intensity led to immediate induction of an event; no signs of epileptiform activity seen; EEG remained unchanged even when intensity reduced and behavioral effects disappeared.
		DBS device turned on and left at same low pulse width (90 μs) but with reduced voltage (5 V).
		Active leads on left stimulator were changed to a more dorsal position to match the position of right stimulator active leads; voltage slowly titrated up to 7.5 V bilaterally over several weeks.
	No side effects have since been reported but depressive symptoms worsened and the patient lost initial beneficial response to DBS.	

Mr. N.’s psychiatric history started around the age of 20 in the form of a depressed mood with severe neurovegetative symptoms and panic attacks without agoraphobia. After the age of 25, the panic attacks resolved with medication, but he remained depressed and anxious. Early in his disease history, he started abusing alcohol to “self-medicate,” per his report, developing significant dependency in his late 20 s. Through the course of his illness, he had a total of four psychiatric admissions and never attempted suicide. The current major depressive episode began in the early 1990s and he tried multiple medications without relief, including Fluoxetine, Paroxetine, Venlafaxine, Mirtazapine, Buproprion, Isocarboxazid, Tranylcypromine, and augmentation strategies including Buspirone, Lithium, Olanzapine, Thioridazine, and Liothyronine. He also failed outpatient Cognitive Behavioral Therapy administered by an experienced Ph.D. psychologist. The only medication that showed a good response was Phenelzine, but the effects faded after some years. In 2001, he had a Vagus Nerve Stimulator (VNS) implanted in the context of a clinical trial, and although the initial response was positive, he relapsed despite gradual increases in stimulation intensity. In 2005, he required a psychiatric admission to receive Electroconvulsive Therapy (ECT). He tolerated the first two sessions with mild post-ECT confusion, but after the 3rd session, he became markedly delirious. The confusion lasted for at least 5 days, at which point it was decided to terminate ECT treatment due to poor tolerability. Given the lack of efficacy, the VNS was explanted in 2006. Later that year he was enrolled in a DBS trial and had a stimulator implanted without complications and with good tolerability. See Malone et al. (2009) for details of the surgical procedure and study protocol. The treatment worked well initially and although he did not remit, he responded positively for approximately 2 years. During the 4–5 months before these events though, his depressive symptoms worsened again and he resumed drinking alcohol, up to four mixed drinks per day. The home medications at the time of admission were Phenelzine 15 mg four times a day, Alprazolam 0.5 mg four times a day, Atorvastatin 10 mg daily, and Omeprazole 20 mg daily.

The day before the admission, Mr. N. called the psychiatric neurotherapeutics team complaining of 2 weeks of bizarre transient episodes, lasting less than 1 min each, in which he was witnessed to suddenly engage in mumbled foul speech with occasional right leg twitching. The frequency and duration of these episodes increased progressively. In one of these episodes, the patient was speaking to his sister when he abruptly interrupted a normal conversation by raising his voice, stuttering incoherent random syllables, and swearing profusely, while also tapping his right leg. In a different event, Mr. N. was talking on the phone when he suddenly started a stuttered conversation with mumbled incoherent words but clear intense swearing. Coprolalia often interrupted speech and behavior, and it generally included a limited repertoire of swear words, though not always following a stereotyped pattern. It is significant that after these episodes, Mr. N. seemed anxious but did not remember what had happened. When he explained these episodes he did it according to what he had been told had occurred, but he had no episodic memories. That said, he was aware of a rush of sympathetic activation, psychological anxiety, and a negative emotional gestalt that he could not relate to concrete events. He never lost consciousness, had generalized or bilateral abnormal movements, or became disoriented. His post-event confusion seemed primarily related to transient amnesia in the context of sympathetic activation, his inability to contextualize the sudden anxiety and negative emotions, and the incapacity to make logical sense of what people described had just happened. Otherwise, he was alert and oriented.

After describing these new symptoms on the phone, the patient was advised to turn the stimulator off and present for evaluation. He was seen in the clinic, DBS parameters were Voltage 7 V (left) and 5 V (right), pulse width 210 μs, frequency 130 Hz, electrode configuration 0 + 1−. He was referred to the Emergency Department that same day, from where he was admitted to the Neurology inpatient service. The patient presented with a normal neurological exam, except for bilateral lower extremity paresthesias which were chronic. He had not suffered any further episodes since turning the stimulator off. Toxicological screens including urine drug screen and blood alcohol were also normal.

The patient was worked up for a possible transient ischemic attack (TIA) or stroke with a head CT, CT angiography of the head and neck, transthoracic echocardiogram, and 24 h Holter, all of which were normal. The patient was initially started on 81 mg of Aspirin daily and continued on his home Atorvastatin, but given all the negative results a TIA was ruled out and Aspirin was discontinued.

**Figure 2:**
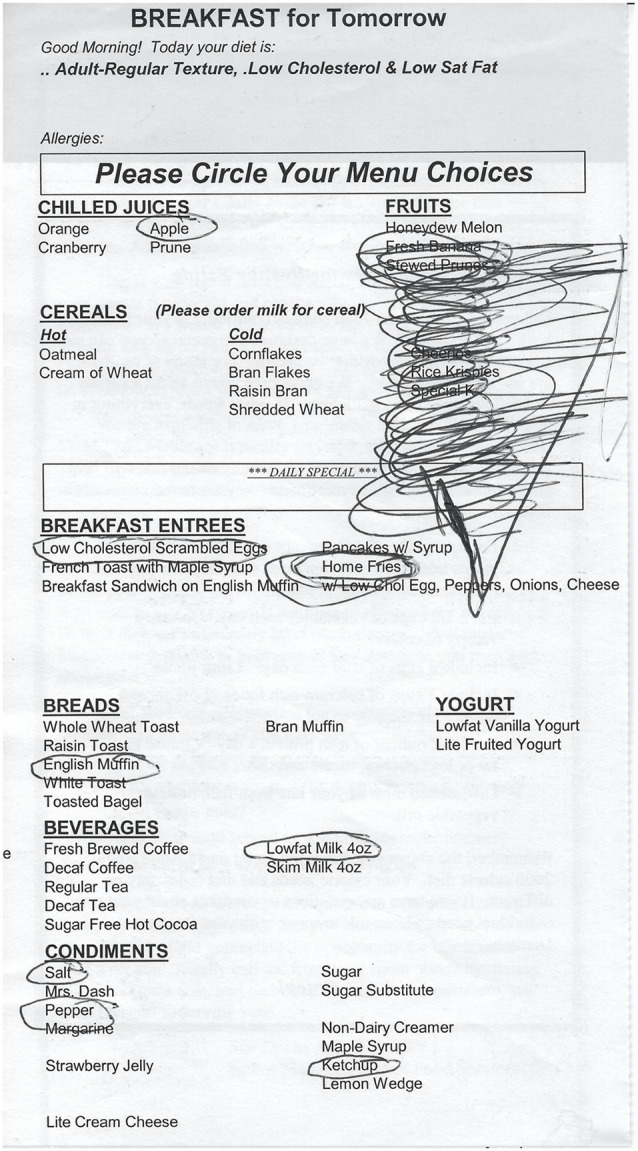
Behavioral and motor manifestation. Drawings made by the patient on a hospital menu during one of the events with right-hand movements.

Given the recurrent brief nature of these events, the patient was also worked up for seizures. An electroencephalogram (EEG) was obtained while the DBS was off and also when it was on: both were normal. The patient was subsequently studied with a 48 h long-term monitoring EEG, with video only for the first 24 h. Two possible but unclear events occurred during this time and the EEG showed no epileptiform activity. At that point, the stimulator was restarted at its original settings, which prompted new spells. The initial events were short and discrete, consisting of abrupt onset of progressively faster and louder mumbled stuttering with clear coprolalia. He also presented right-sided tic-like motor automatisms (right hand or foot abrupt-onset jerk-like movements or tapping-like behaviors that were more complex and not as fast as myoclonus) and an egodystonic “rush” of physical and psychological activation, “as if I were fighting with myself inside my head.” The patient had minimal awareness or memory of the events after they occurred but knew one had just happened. He was always conscious and interactive though, before, during, and after the events. After the events, he was alert and oriented, but mildly confused and lacking a good recollection of the details. He had approximately six events per day while the stimulator was on. During one of the events, as he was completing his breakfast preferences in the hospital menu, an episode started and he continued drawing circles on the paper in what seemed to be a mix of jerk-like movements and transient perseveration (Figure 2).

At this point and with a negative diagnostic workup, the psychiatry neurotherapeutics team was consulted. To clarify a possible iatrogenic effect from the DBS stimulator, the voltage of the stimulator was increased from 7 to 8 V. A few minutes later the voltage was further increased to 8.5 V and an event started immediately. The stimulator voltage was then reduced back to 7 V and the event stopped abruptly. In the light of the previous negative workup and the observed dose-related response of the spells, an iatrogenic etiology related to DBS became the most likely explanation. The patient was discharged with the stimulator off and a follow-up appointment with the neurotherapeutics team the next day. No changes were made to his medications. He did not present any signs of alcohol withdrawal.

The patient presented for follow up. The stimulator was turned on and the left electrode was set to provide a lower charge density by decreasing the pulse width to 90 μs. Acutely, the patient described no side effects. Three weeks later though, he called to let us know he had had a few brief similar episodes. He was then advised to turn the stimulator off. He was seen a few days later to do an outpatient EEG with the stimulator on. When the patient was in the neurophysiology unit, the DBS voltage was increased eliciting an event. The EEG was carefully analyzed with particular emphasis on the period when the event was noted, but no signs of epileptiform activity were observed. The behavioral effects disappeared as the voltage was reduced, but the EEG remained unchanged. After this assessment, the stimulator was left at the same low pulse width (90 μs) but with reduced voltage (5.0 V).

On closer radiographic analysis of DBS electrodeposition, the left stimulator was observed to be more ventral and rostral than the right (Figure 1B). The active leads on the left stimulator were therefore changed to a more dorsal position, from 0 + 1− to 1 + 2−. In the following weeks, the voltage was slowly titrated up with close monitoring of mood symptoms and DBS tolerability in several outpatient visits. The voltage was increased sequentially up to 7.5 V bilaterally, but the pulse width and active leads were unchanged. No side effects were reported since, but the patient’s depressive symptoms worsened and he lost the initial antidepressant benefit: in the first year after DBS implantation, Montgomery-Asberg Depression Rating Scale (MADRS) scores improved from 36 to 19 points, slightly worsening at the end of year 2 (8 months before these events) up to 23 points. After this event and with the new DBS settings, depression symptoms continued to worsen with a MADRS of 32 points at the end of year 3 (4 months after these events) and 36 points at the end of year 4.5, the last recorded score. The patient was eventually explanted given lack of efficacy, poor compliance with follow-up visits, and new medical comorbidities that could be more effectively and safely monitored using MRI without the limitations of a metallic foreign body.

### Positron Emission Tomography Protocol

In the context of a neuroimaging research study, previous to the development of these symptoms, Mr. N. was scanned with Positron Emission Tomography (PET) to measure brain perfusion. We analyzed these data for him individually, aiming to identify specific brain perfusion patterns associated with the iatrogenic DBS location.

Images were acquired using a 15-slice whole-body tomography scanner (model 4096; Scanditronix, General Electric, Milwaukee, WI, USA) in stationary mode. The slice geometry consisted of contiguous slices with a center-to-center distance of 6.5 mm (axial field 97.5 mm) and an axial resolution of 6 mm full-width at half-maximum. Head alignment was made relative to the canthomeatal line. Once the head was in place, an overlying face mask attached to a vacuum and a nasal cannula which delivered the [O–15]-CO_2_ (concentration 2,960 MBq/L; flow rate 2 L/min) was positioned.

Eight runs (with two runs of four conditions) were performed: (1) DBS off; (2) DBS on in monopolar configuration at contact 1; (3) DBS on in monopolar configuration at contact 3; and (4) DBS on in bipolar configuration between contacts 0(+) and 1(−). A 10 min rest period was imposed between each successive PET run, to allow for decay of O–15 radiation signal.

### Data Analysis

The PET images were preprocessed using SPM5. We compared perfusion patterns when DBS was on in the bipolar configuration that induced these events (0 + 1−) and when DBS was on in monopolar configuration at contact 3, more dorsally like the post-discharge montage that did not elicit aberrant behaviors. Based on previous studies of GTS, we restricted our search territory to *a priori* regions of interest [the anterior cingulate, basal ganglia (caudate, putamen, pallidum), and insula] as defined by the Wake Forest University Pick Atlas (Maldjian et al., 2003). To correct for multiple comparisons, a false discovery rate (FDR) correction was applied with a significance threshold of *p* < 0.05.

## Results

**Figure 3:**
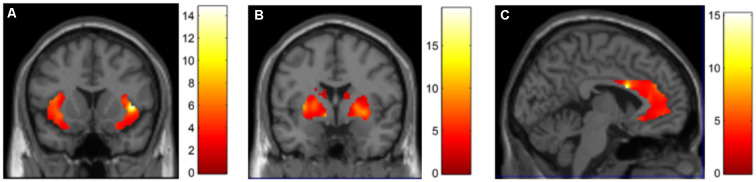
DBS-evoked changes in brain perfusion with 15O CO2 PET. Contrasting DBS on at contact 0 + 1 (bipolar) > contact 3 (unipolar), we identified increases in perfusion in the **(A)** insula, **(B)** basal ganglia and **(C)** anterior cingulate.

DBS stimulation with the iatrogenic montage (0 + 1−) compared to stimulation with the monopolar configuration at contact 3 led to a significant increase in perfusion in the insula (peak cluster MNI coordinates = 42, 14, 2, *k* = 1,646 voxels, *Z*-score = 4.21, FDR corrected *p* < 0.05, Figure 3A), pallidum (peak cluster MNI coordinates = −8, 2, −4, *k* = 2,005 voxels, *Z*-score = 4.50, FDR corrected *p* < 0.05, Figure 3B), and the anterior cingulate (peak cluster MNI coordinates = −4, 10, 24, *k* = 2,494 voxels, *Z*-score = 4.24, FDR corrected *p* < 0.05, Figure 3C).

## Discussion

We report the case of a patient with treatment-resistant MDD who after 32 months of moderately effective DBS to the VC/VS presented with recurrent, brief, and discrete episodes of abrupt-onset and progressively faster and louder coprolalia with stuttered mumbled speech; tic-like motor behavior in his right arm and leg; rushes of anxiety, angry prosody, angry affect; and transient amnesia without confusion. These episodes could be replicated in a montage- and voltage-dependent manner, stimulating the most ventral electrodes at higher voltages, while more dorsal stimulation and/or lower voltage did not elicit these episodes and even terminated ongoing events.

The diagnostic workup ruled out cerebrovascular etiologies such as TIAs. The recurrent and episodic presentation in the context of electrical brain stimulation was suggestive of seizures. Complex partial seizures can present with coprolalia, particularly when affecting the frontal lobes (Daniel and Perry, 2016; Massot-Tarrús et al., 2016; Xu et al., 2020). It was unclear if the patient had premonitory urges or was able to suppress the motor symptoms or coprolalia, as he did not remember the events: this may have helped distinguish tics from motor stereotypies or complex motor seizures (Robertson et al., 2017).

The cognitive changes after the events were suggestive of generalized or complex partial seizures, but further examination revealed that it was not confusion as one may typically see in post-ictal states, but brief transient episodic amnesia leading to a discontinuous perception of both external and internal events. Although certain aspects of the phenomenology were indeed suspicious for complex partial seizures and deep frontal epileptic sources may be poorly detected in surface EEG, video-EEG recordings during several active episodes (including lateralized motor phenomena) did not identify epileptic activity despite careful analysis by the Epilepsy team. While these stimulation-dependent symptoms should be electrical, they did not seem to stem from epileptic patterns of neuronal firing, but more likely, from the non-epileptic artificial activation of circuits involved in cognition, behavior, affect, and movement.

It is reasonable to consider if the abnormal movements were indeed tics or stereotypies, but the co-occurrence of abrupt onset coprolalia that interrupts the flow of normal conversation (like in GTS) and does not seem to respond to pain or other factors that could trigger cursing led us to hypothesize that we were activating a circuit of regions similar to that involved in GTS, and our analysis of the PET perfusion data seemed to confirm this hypothesis. In this context, we favored the use of tic-like (not tics) to describe the abnormal movements, though we acknowledge the symmetries with stereotypies.

The PET results support the hypothesis that maladaptive DBS activation of functional circuits caused these events: DBS of the ventral-anterior VC/VS (which elicited the aberrant behaviors) led to increased perfusion in a circuit of regions involving the insula, basal ganglia, and the anterior cingulate, compared to a more dorsal VC/VS configuration that did not elicit symptoms. Indeed, GTS has been associated with hyperperfusion in the striatum and anterior cingulate (Robertson et al., 2017). Further support for this iatrogenic hypothesis is given by the parameter-dependent nature of these symptoms: they were elicited when the stimulator was turned on, and remitted with lower voltages or switching to dorsal electrode positions or off. Of note, iatrogenic GTS symptoms have been reported with other therapies as well: Lamotrigine, an anticonvulsant and mood stabilizer, has been described to trigger vocal tics in an adult patient (Seemüller et al., 2006), and motor and vocal tics in five children (Sotero de Menezes et al., 2000). While our evidence does not offer answers regarding the role of white matter tracts, connections exist linking these regions and integrating motor control, cognitive and emotional processing (Testini et al., 2016), which have been proven effective for GTS and other neuropsychiatric syndromes when therapeutically modulated with DBS (Marano et al., 2019). We should note that while the clinical phenotype of GTS is heterogeneous, the symptoms we reported were Tourette-like but did not perfectly mirror the symptomatology observed in idiopathic GTS.

Two factors seemed to be related to the parameter-dependent aberrant behaviors: higher voltages and more ventral electrodes on the left side. Careful examination of neuroradiological images identified that the left electrode was slightly more ventral and more anterior than the right. This led to the clinical decision to move to more dorsal electrode configurations which resolved the Tourette-like events, but unfortunately also the antidepressant benefit. The VC/VS is a complex region that includes several behaviorally relevant white matter tracts and subcortical gray matter structures. Such dense functional and structural diversity could explain how small spatial variations in electrode placement or electric field size and topography may lead to unintended physiological activation of circuits leading to maladaptive behavior, cognition, affect and movement, instead of the intended therapeutic adaptive effects. This case highlights the critical importance of a careful understanding of the patient-specific functional anatomy of DBS targets and the use of individualized strategies guided by imaging, physiology, or both for target selection, not only to optimize efficacy but also to avoid complications. Complications which, in functional neurosurgery, are not always traditional surgical side-effects such as bleeding, infection, trauma, etc., but can also result, like in our patient and others (Widge et al., 2016), from the maladaptive activation of behaviorally-relevant nodes and circuits.

## Data Availability Statement

The datasets presented in this article are not readily available.

## Ethics Statement

The studies involving human participants were reviewed and approved by Partners Healthcare IRB. The patients/participants provided their written informed consent to participate in this study. Written informed consent was obtained from the individual(s) for the publication of any potentially identifiable images or data included in this article.

## Author Contributions

JAC and DDD led the clinical management. TC analyzed the PET data. JAC wrote the main draft of the manuscript. All authors contributed to the article and approved the submitted version.

## Conflict of Interest

DDD has received honoraria, consultation fees and/or royalties from Medtronic, Wyeth, Jazz Pharmaceuticals, Bristol Myers Squibb, Brand Ideas, and Reed Elsevier. The authors declare that the research was conducted in the absence of any commercial or financial relationships that could be construed as a potential conflict of interest.

## References

[B1] Andrade P. Visser-Vandewalle V. (2016). DBS in Tourette syndrome: where are we standing now? J. Neural Transm. 123, 791–796. 10.1007/s00702-016-1569-7 27209036

[B2] Arulpragasam A. R. Chou T. Corse A. K. Kaur N. Deckersbach T. Camprodon J. A. (2013). Future directions of deep brain stimulation: current disorders, new technologies. Psychiatr. Ann. 43, 366–373. 10.3928/00485713-20130806-05

[B3] Daniel C. Perry M. S. (2016). Ictal coprolalia: a case report and review of Ictal speech as a localizing feature in epilepsy. Pediatr. Neurol. 57, 88–90. 10.1016/j.pediatrneurol.2015.11.013 26880529

[B4] Herrington T. M. Cheng J. J. Eskandar E. N. (2016). Mechanisms of deep brain stimulation. J. Neurophysiol. 115, 19–38. 10.1152/jn.00281.2015 26510756 PMC4760496

[B5] Kaur N. Chou T. Arulpragasam A. R. Corse A. K. Deckersbach T. Evans K. C. (2013). Deep brain stimulation for treatment-resistant depression. Psychiatr. Ann. 43, 358–365. 10.3928/00485713-20130806-04

[B6] Maldjian J. A. Laurienti P. J. Kraft R. A. Burdette J. H. (2003). An automated method for neuroanatomic and cytoarchitectonic atlas-based interrogation of fMRI data sets. NeuroImage 19, 1233–1239. 10.1016/s1053-8119(03)00169-1 12880848

[B7] Malone D. A. Jr. Dougherty D. D. Rezai A. R. Carpenter L. L. Friehs G. M. Eskandar E. N.. (2009). Deep brain stimulation of the ventral capsule/ventral striatum for treatment-resistant depression. Biol. Psychiatry 65, 267–275. 10.1016/j.biopsych.2008.08.029 18842257 PMC3486635

[B8] Marano M. Migliore S. Squitieri F. Insola A. Scarnati E. Mazzone P. (2019). CM-Pf deep brain stimulation and the long term management of motor and psychiatric symptoms in a case of Tourette syndrome. J. Clin. Neurosci. 62, 269–272. 10.1016/j.jocn.2018.12.029 30612913

[B9] Massot-Tarrús A. Mousavi S. R. Dove C. Hayman-Abello S. S. Hayman-Abello B. Derry P. A.. (2016). Coprolalia as a manifestation of epileptic seizures. Epilepsy Behav. 60, 99–106. 10.1016/j.yebeh.2016.04.040 27195785

[B10] Robertson M. M. Eapen V. Singer H. S. Martino D. Scharf J. M. Paschou P.. (2017). Gilles de la Tourette syndrome. Nat. Rev. Dis. Primers 3:16097. 10.1038/nrdp.2016.97 28150698

[B11] Seemüller F. Dehning S. Grunze H. Müller N. (2006). Tourette’s symptoms provoked by lamotrigine in a bipolar patient. Am. J. Psychiatry 163:159. 10.1176/appi.ajp.163.1.159 16390908

[B12] Sotero de Menezes M. A. Rho J. M. Murphy P. Cheyette S. (2000). Lamotrigine-induced tic disorder: report of five pediatric cases. Epilepsia 41, 862–867. 10.1111/j.1528-1157.2000.tb00254.x 10897158

[B13] Testini P. Min H.-K. Bashir A. Lee K. H. (2016). Deep brain stimulation for Tourette’s syndrome: the case for targeting the thalamic centromedian-parafascicular complex. Front. Neurol. 7:193. 10.3389/fneur.2016.00193 27891112 PMC5102892

[B14] Widge A. S. Licon E. Zorowitz S. Corse A. Arulpragasam A. R. Camprodon J. A.. (2016). Predictors of hypomania during ventral capsule/ventral striatum deep brain stimulation. J. Neuropsychiatry Clin. Neurosci. 28, 38–44. 10.1176/appi.neuropsych.15040089 26404172 PMC5770191

[B15] Xu C. Ma K. Zhang X. Yu T. Zhang G. Wang Y.. (2020). Ictal coprolalia occurs due to the activation of the temporal-orbitofrontal network in patients with epilepsy. J. Neurol. Sci. 409:116634. 10.1016/j.jns.2019.116634 31864073

